# Targeting ADT-Induced Activation of the E3 Ubiquitin Ligase Siah2 to Delay the Occurrence of Castration-Resistant Prostate Cancer

**DOI:** 10.3389/fonc.2021.637040

**Published:** 2021-04-16

**Authors:** Tingmang Yan, Dapeng Zhou, Youwei Shi, Di Cui, Juntao Jiang, Bangmin Han, Shujie Xia, Zhou Wang, Haitao Liu, Wenhuan Guo, Yifeng Jing

**Affiliations:** ^1^ Department of Urology, Shanghai General Hospital, Shanghai Jiao Tong University School of Medicine, Shanghai, China; ^2^ Department of Urology, University of Pittsburgh School of Medicine, Pittsburgh, PA, United States; ^3^ Department of Pharmacology and Chemical Biology, University of Pittsburgh School of Medicine, Pittsburgh, PA, United States; ^4^ Department of Pathology, University of Pittsburgh School of Medicine, Pittsburgh, PA, United States; ^5^ UPMC Hillman Cancer Center, University of Pittsburgh School of Medicine, Pittsburgh, PA, United States; ^6^ Pathology Center, Shanghai General Hospital/Faculty of Basic Medicine, Shanghai Jiao Tong University School of Medicine, Shanghai, China

**Keywords:** castration-resistant prostate cancer, androgen receptor, androgen deprivation therapy, Siah2, E3 ubiquitin ligase

## Abstract

Siah2 is an E3 ubiquitin ligase that targets androgen receptor (AR) and plays an important role in the development of castration-resistant prostate cancer (CRPC). However, the regulation of Siah2 in prostate cancer (PCa) is largely unknown. In this study, we used AR-dependent and -independent cells lines to investigate the cellular roles of AR and androgen deprivation therapy (ADT) on Siah2 protein levels and E3 ligase activity using Western blotting and co-immunoprecipitation. We also validated our findings using patient samples taken before and after ADT. Finally, we used xenograft tumor models to test the effects of ADT combined with vitamin K3 (Vit K3) on tumor growth *in vivo*. Our results showed that AR stabilizes Siah2 protein by attenuating its self-ubiquitination and auto-degradation, likely by blocking its E3 ubiquitin ligase activity. Conversely, ADT decreased Siah2 protein expression but enhanced its E3 ligase activity in PCa cells. Notably, the findings that ADT decreasing Siah2 protein expression were verified in a series of paired PCa samples from the same patient. Additionally, we found that ADT-induced Siah2 activation could be abolished by Vit K3. Strikingly, ADT combined with Vit K3 treatment delayed the occurrence of CRPC and dramatically inhibited the growth of tumor xenografts compared with ADT treatment alone. AR is an inhibitor of Siah2 in PCa, and ADT leads to the continuous activation of Siah2, which may contribute to CRPC. Finally, ADT+Vit K3 may be a potential approach to delay the occurrence of CRPC.

## Introduction

Prostate cancer (PCa) is the most commonly diagnosed malignancy and the second leading cause of cancer-related mortality in American men, with an estimated 174,650 new cases and 31,620 deaths expected in 2020 (1). Notably, PCa has also become the most common male urogenital malignancy in China (2). Androgen deprivation therapy (ADT) remains the first-line therapy for men with metastatic PCa; however, most patients ultimately relapse with castration-resistant prostate cancer (CRPC), which is currently incurable and accounts for most PCa-associated mortalities (3). Although the mechanisms of CRPC remain unclear, accumulating evidence has shown that androgen receptor (AR) signaling is central to CRPC progression (4, 5). Several mechanisms have been suggested to mediate androgen-independent AR signaling, including AR mutations, AR gene amplifications or overexpression, expression of specific AR splice variants, intratumoral androgen production, and abnormal post-translation modification of AR, such as phosphorylation, methylation, acetylation, ubiquitination, and SUMOylation (6).

Siah2 is a RING finger type ubiquitin ligase comprising a catalytic RING domain, two zinc fingers, and a C-terminal substrate-binding domain (SBD) (7). Many proteins have been identified as substrates of Siah2, including N-CoR, PHD, Sprouty2, β-catenin, and AR (8–12). Several studies have shown that Siah2 has important roles in tumorigenesis and metastasis in multiple cancers, including breast cancer, lung cancer, pancreatic cancer, melanoma, and PCa (13). Importantly, Siah2 has been identified as an E3 ubiquitin ligase of AR that specifically targets a selective pool of NCOR1-bound, repressed AR chromatin complexes for degradation. These complexes are typically involved in lipid metabolism, cell motility, and proliferation in PCa cells. Additionally, Siah2 is required for CRPC tumor growth in mice, whereas Siah2 deletion increases the castration sensitivity of TRAMP mice (12). Thus, Siah2 is a critical player in CRPC development.

Given its role in CRPC, it is important to know how Siah2 is regulated in PCa. We and others have previously reported that Siah2 is regulated by proteins, such as DHX15 and AKR1C3 in PCa (14, 15). However, the clinical relevance of these studies still needs further verification. In this study, we show that AR is a substrate of Siah2 that can inhibit Siah2 self-ubiquitination, stabilize Siah2 expression, and decrease its E3 ubiquitin ligase activity in PCa cells. Additionally, ADT significantly reduced Siah2 expression and enhanced its ligase activity. Notably, these findings are closely related to clinical PCa samples. Importantly, treatment with the specific Siah2 inhibitor, vitamin K3 (Vit K3), delayed LNCaP tumor progression to castration resistance in LNCaP tumors. Therefore, Vit K3 might be an adjuvant that can be combined with ADT to treat advanced PCa and delay CRPC.

## Materials and Methods

### Plasmid Constructs

All plasmid constructs were created or obtained as previously described (14).

### Cell Culture and Transfection

The human PCa cell lines LNCaP, 22Rv1, and PC3, and HEK293 cells were obtained from American Type Culture Collection (ATCC, Manassas, VA, USA). Cells were maintained in the appropriate medium (RPMI-1640 for LNCaP, PC3, and 22Rv1 and DMEM for HEK293) supplied with 10% fetal bovine serum, 5% antibiotics, and 1% l-glutamine at 37°C with 5% CO_2_. Cells were verified as mycoplasma free using PCR. Cells were cultured in phenol red-free medium supplied with 5% dextran-coated charcoal-stripped fetal bovine serum (CS-FBS) for 24 h before treatment with dihydrotestosterone (Sigma-Aldrich, St Louis, MO, USA). In some experiments, cells were treated with the protein synthesis inhibitor cycloheximide (Sigma-Aldrich) at 50 μg/ml and/or the proteasome inhibitor MG132 (Sigma-Aldrich) at 5 μM for various times as described in the figure legends. Cell transfection was separately performed with Lipofectamine 2000 (Invitrogen, Carlsbad, CA, USA) for constructs or siRNAs according to the manufacturer’s protocols. The siRNA targeting AR was purchased from Thermo Fisher Scientific (Waltham, MA, USA). The siRNAs targeting Siah2 were as follows: Siah2-1, 5′-UAUGACUUGCUUUCCUAGGCAAUCCAC-3′; Siah2-2, 5′-CCUCCCAUUCCUAACACACUGAUCUAU-3′.

### Western Blot Analysis and Immunoprecipitation

Western blot and immunoprecipitation assays were performed as previously described (14). Primary antibodies against Siah2 (NBP1-19648, Novus Biologicals, Littleton, CO, USA, 1:1000), AR (sc-816, Santa Cruz Biotechnology, Dallas, TX, USA, 1:1000), Sprouty2 (sc-30049, Santa Cruz Biotechnology, 1:1000), Flag M2 (F1804, Sigma-Aldrich, 1:2000), Myc (MMS-150, Covance, Princeton, NJ, USA, 1:2000), HA (MMS-101P, Covance, 1:2000), and Tubulin (abs131993, Absin Bioscience, Shanghai, China, 1:5000) were used in the study.

### *In Vivo* Ubiquitination Assay

HEK293 cells were transfected with Flag-AR, GFP-AR, and HA-ubiquitin as indicated for 24 h, and then were treated with 5 μM MG132 for 16 h before harvest. Cells were lysed in 100 μl RIPA buffer with 1% SDS to disrupt protein–protein interactions, and then boiled for 10 min at 95°C. The lysates were diluted 10-fold with RIPA buffer and immunoprecipitated with Flag M2 gel for 3 h followed by incubation with protein A/G Plus-Agarose for 3 h. After three washes, the immunoprecipitates were subjected to Western blot analysis.

### RT-PCR and Real-Time PCR

RT-PCR and real-time PCR were performed as described previously (14). The primer sequences used were as follows: AR, 5′-TGGATGGATAGCTACTCCGG-3′ and 5′-CCCAGAAGCTTCATCTCCAC-3′; GAPDH, 5′-CGACCACTTTGTCAAGCTCA-3′ and 5′-AGGGGAGATTCAGTGTGGTG-3′; and Siah2, 5′-AGGTTGCCCTCTGCCGATA-3′ and 5′-ACATAGGTGAGTGGCCAAATCTC-3′.

### Immunohistochemistry

Formalin-fixed paraffin-embedded (FFPE) PCa specimens were obtained from the surgical pathology archives of Shanghai General Hospital. Use of these prostate tissues was approved by the Shanghai General Hospital Review Board. Immunohistochemistry was performed as previously described (14) using an anti-Siah2 antibody (NB110-88113 [24E6H3], Novus Biologicals, 1:500).

### Transwell Migration and BrdU Incorporation Assays

The transwell migration and BrdU incorporation assays were performed as previously described (14).

### Animals and Xenograft Tumors

Male athymic BALB/c nude mice (5–6 weeks old) purchased from the Animal Center of the Chinese Academy of Sciences (Shanghai, China) were subcutaneously injected in one flank with 300 μl of LNCaP cells (1 × 106) mixed 1:1 (v:v) with Matrigel (Invitrogen). Tumors were measured with calipers twice per week. Tumor volumes were calculated using the formula length × width 2 × 0.52. Mice were randomized into three groups once the tumor volume reached 0.6 mm3: the sham-operated (n=5), castration (n=5), and castration+Vit K3(Sigma-Aldrich) injection groups (n=5). For the latter, a dose of 10 mg/kg Vit K3 (dissolved in DMSO at a final concentration of 0.1%) was administered *via* twice weekly intra-peritoneal injections. Tumor growth was monitored twice per week for 7 weeks, at which point the mice were sacrificed and tumors were harvested for Western blot analyses. All animal studies were conducted in accordance with the Shanghai Jiao Tong University Medical School’s Animal Committee guidelines.

### Statistical Analysis

Data are presented as mean ± standard error (SEM) or mean ± standard deviation (SD). Statistical analyses were performed with Student’s t-test or one-way ANOVA. *P* < 0.05 was considered statistically significant.

## Results

### Androgens Stabilized Siah2 Protein in PCa Cells

**Figure 1:**
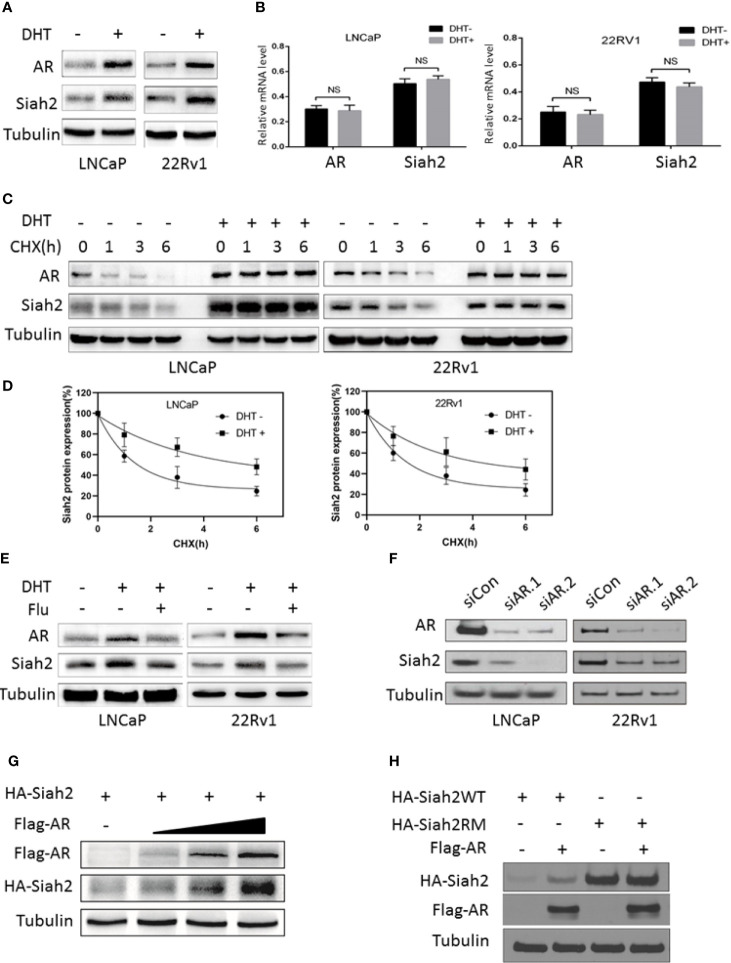
Androgens stabilized Siah2 protein levels. **(A)** LNCaP and 22Rv1 cells were cultured in CS-FBS medium for 24 h, and then treated with 10 nM DHT or vehicle for 24 h. AR and Siah2 protein expression were detected using Western blotting. **(B)** LNCaP and 22Rv1 cells were treated as described in A, and then AR and Siah2 mRNA expression were detected by qPCR. **(C)** LNCaP and 22Rv1 cells were treated as described in A, and then treated with cycloheximide (CHX, 50 μg/ml) for 1, 3, and 6 h, after which cell lysates were subjected to Western blotting. **(D)** Degradation curves of Siah2 by cycloheximide chase experiments in the presence or absence of DHT. **(E)** LNCaP and 22Rv1 cells were treated as described in A, and then treated with flutamide (5 μM) for 24 h. AR and Siah2 protein expression were detected using Western blotting. **(F)** LNCaP and 22Rv1 cells were transfected with two different siRNAs targeting Siah2 or control for 72 h, and then subjected to Western blotting to detect Siah2 and AR expression. **(G)** HEK293 cells were transfected with HA-Siah2 and different doses of Flag-AR for 48 h. Cell lysates then were subjected to Western blotting and probed with anti-Flag and anti-HA antibodies. **(H)** 293 cells were transfected with Flag-AR and HA-Siah2WT or HA-Siah2RM for 48 h, cell lysates were subjected to Western blotting and probed with anti-Flag and anti-HA antibodies. NS, non-significant.

Due to the pivotal role of Siah2 in the development of CRPC, here we asked how Siah2 was regulated during ADT. Therefore, we first assessed whether Siah2 expression was altered following the treatment of PCa cells with androgens. Notably, treatment of LNCaP cells with 10 nM dihydrotestosterone (DHT) for 24 h led to a significant increase in Siah2 protein expression (**Figure 1A**). We next determined the expression of Siah2 in response to DHT in another AR-positive PCa cell line, 22Rv1. Treating 22Rv1 cells with DHT also dramatically increased Siah2 expression (**Figure 1A**). Furthermore, DHT effect on Siah2 showed a dose-dependent manner (**Figure S1A**). Nevertheless, DHT did not alter Siah2 mRNA levels in either LNCaP or 22Rv1 cells, which suggests that DHT regulates Siah2 expression at the post-transcriptional level (**Figure 1B**). To determine whether androgens altered Siah2 stability, we monitored the half-life of endogenous Siah2 in the presence of the protein synthesis inhibitor cycloheximide; 10 nM DHT prolonged the half-life of Siah2 from approximately 1.5 to 5 h in both LNCaP and 22Rv1 cells (**Figures 1C, D** **)**. To further address the role of AR in DHT-mediated Siah2 stabilization in PCa cells, siRNAs specifically targeting AR or the antagonist flutamide were used to block AR. Both siRNAs and flutamide decreased Siah2 protein expression (**Figures 1E, F** **)**. Consistently, DHT treatment increases Siah2 protein level in AR-positive LNCaP cells but not in AR-negative PC3 cells (**Figure S1B**). Additionally, co-transfection of HA-Siah2 with different amounts of Flag-AR into HEK293 cells showed that Siah2 protein levels increased with increasing amount of exogenous AR expression (**Figure 1G**). Finally, we found AR increased the expression of Wild-type Siah2 (Siah2-WT) but not RING mutant Siah2 (Siah2-RM, which lacks ubiquitin ligase activity), suggesting that AR stabilizes Siah2 by inhibiting its ubiquitin ligase activity (**Figure 1H**). Together, these findings demonstrate that AR stabilizes Siah2 protein in PCa cells.

### AR Inhibits Siah2 Self-Ubiquitination and Decreases Its E3 Ligase Activity

**Figure 2:**
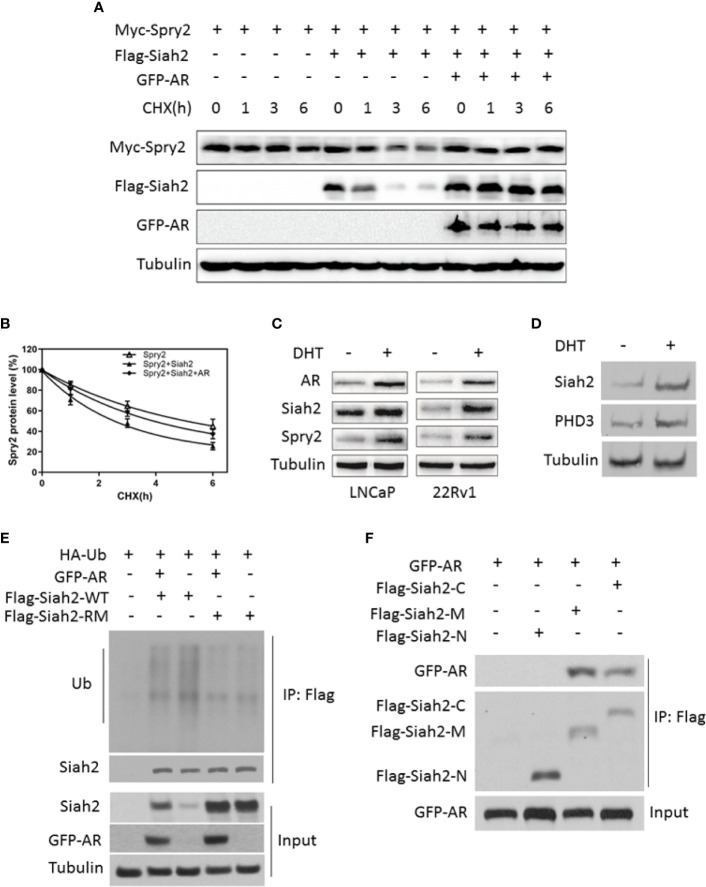
AR inhibited Siah2 auto-ubiquitination and decreased its E3 ligase activity. **(A)** HEK293 cells were transfected with Flag-Siah2, GFP-AR, and Myc-Spry2 as indicated for 48 h, and then treated with cycloheximide (CHX, 50 μg/ml) for 1, 3, and 6 h. Cell lysates were then subjected to Western blotting. **(B)** Degradation curves of Spry2 by cycloheximide chase experiments in the presence or absence of Siah2 or AR. **(C)** LNCaP and 22Rv1 cells were cultured in CS-FBS medium for 24 h, and subsequently treated with 10 nM DHT or vehicle for 24 h. Spry2, Siah2, and AR protein expression were detected by Western blotting. **(D)** LNCaP cells were cultured in CS for 24 h, subsequently treated with 10 nM DHT or vehicle for 24 h. Siah2and PHD3 protein expression were detected by Western blotting. **(E)** PC3 cells were transfected with Flag-Siah2-WT or Flag-Siah2-RM, GFP-AR and HA-Ub as indicated for 24 h, and treated with 5 μM MG132 for 16 h. Cell lysates were immunoprecipitated with Flag antibody and subjected to Western blotting with HA and Flag antibody. **(F)** GFP-AR was co-transfected with Flag-Siah2 fragments (N, N-terminal region; M-middle region; C, C-terminal region) into HEK293 cells. Cell lysates were immunoprecipitated with Flag antibody and subjected to Western blotting with Flag and GFP antibodies.

Like other RING finger E3 ubiquitin ligases, Siah2 limits its own expression by self-ubiquitination and auto-degradation, which is a sign of its ubiquitin-ligase activity. To test the effect of AR stabilization on Siah2 E3 ligase activity, we monitored Siah2-mediated degradation of Sprouty2 (Spry2), one of the classic substrates of Siah2 and a marker for Siah2 ligase activity. Overexpressing wide-type Siah2 effectively reduced Spry2 half-life from approximately 5 to 3 h, while co-expression of AR prolonged Spry2 half-life to approximately 4 h (**Figures 2A, B** **)**. RING mutant Siah2 alone or co-expression with AR did not change the half-life of Spry2 (**Figure S2A**), which indicated AR inhibits Siah2 E3 ligase activity. We next assessed the effect of DHT on endogenous Spy2 and PHD3, another classic substrate of Siah2. Treatment with 10 nM DHT for 24 h resulted in significantly increased Spry2 and Siah2 levels in both LNCaP and 22Rv1 cells (**Figure 2C**). As expected, DHT treatment increased PHD3 expression as well (**Figure 2D**). These observations suggest that Siah2 ligase activity was inhibited in the presence of AR.

We next determined whether AR stabilized Siah2 protein by inhibiting its self-ubiquitination. Therefore, we co-expressed Flag-Siah2-WT or Flag-Siah2-RM, HA-Ub, and GFP-AR in PC3 cells and treated the cells with the proteasome inhibitor MG132 for 6 h. We then immunoprecipitated Flag-Siah2 using anti-Flag M2 beads and performed Western blot analysis with an anti-HA antibody to detect Flag-Siah2 ubiquitination levels. As expected, Siah2-WT ubiquitination was decreased in the presence of AR, while ubiquitination of Siah2-RM was not changed (**Figure 2E**). Siah2 comprises three different domains: N-terminal domain, central RING domain/zinc finger domain, and C-terminal SBD. To map the Siah2 domains required for this AR interaction, we generated truncation mutants of Siah2 (14) and co-transfected them individually with AR into HEK293 cells. These co-immunoprecipitation assays revealed that both the SBD and the central RING domain/zinc finger domains were required for the interaction with AR (**Figure 2F**).

### ADT Decreased Siah2 Expression and Increased Its Ligase Activity

**Figure 3:**
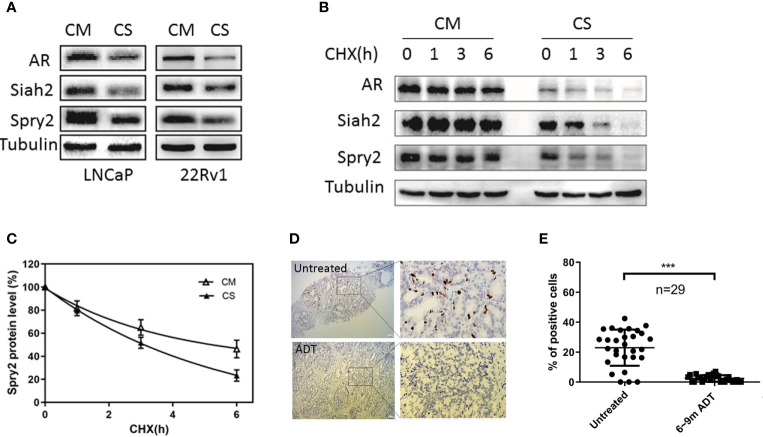
ADT decreased Siah2 expression and increased its ligase activity. **(A)** LNCaP and 22Rv1 cells were cultured in complete medium for 24 h, and then maintained in CS-FBS medium for another 24 h. Spry2, Siah2, and AR protein expression were detected by Western blotting. **(B)** LNCaP cells were treated as described in **(A)**, and then treated with cycloheximide (CHX, 50μg/ml) for 1, 3, and 6 h, after which cell lysates were subjected to Western blotting. **(C)** Degradation curves of Siah2 by cycloheximide chase experiments. **(D)** Representative images of Siah2 immunohistochemistry staining in PCa specimens from the same patient. **(E)** Quantification of Siah2 immunohistochemistry staining in PCa samples from 29 patients who underwent radical prostatectomy after between 6 and 9 months of ADT treatment. ***P < 0.001.

Given that AR stabilized Siah2 protein and inhibited its E3 ligase activity, we hypothesized that ADT may increase Siah2 activity in PCa. To test the effect of ADT on Siah2 in PCa cells, LNCaP or 22Rv1 cells were cultured in medium supplemented with 5% CS-FBS (to mimic ADT conditions) or in complete medium. As expected, both Siah2 and Spry2 expressions were significantly decreased when the cells were cultured in CS-FBS medium compared with those in complete medium, which was consistent with AR expression (**Figure 3A**). This result suggested that ADT treatment decreased Siah2 expression while enhancing its E3 ligase activity. To further confirm the effect of ADT on Siah2 activity, we monitored endogenous Spry2 and Siah2 expression in LNCaP cells using the cycloheximide chase assay. The Spry2 half-life was reduced from approximately 4.5 h in cells cultured in complete medium to 3 h when cells cultured in CS-FBS medium (**Figures 3B, C** **)**, which suggests increased Siah2 activity.

To assess the relevance of our findings in human PCa, we evaluated Siah2 protein expression in 29 PCa patients who underwent radical prostatectomy after ADT treatment for 6–9 months. Siah2 was detected immunohistochemically both in biopsy samples (before ADT) and in radical prostatectomy samples (after ADT) from the same patient. Siah2 showed a nuclear expression pattern as described previously (14). Notably, Siah2 staining was significantly reduced in all patients after ADT (**Figures 3D, E** **)**, which was consistent with our *in vitro* findings. These data suggest that ADT decreases Siah2 protein expression and enhances its E3 ligase activity in PCa.

### Vit K3 Attenuated the Effects of ADT on Siah2 and Inhibited the Growth and Motility of PCa Cells

**Figure 4:**
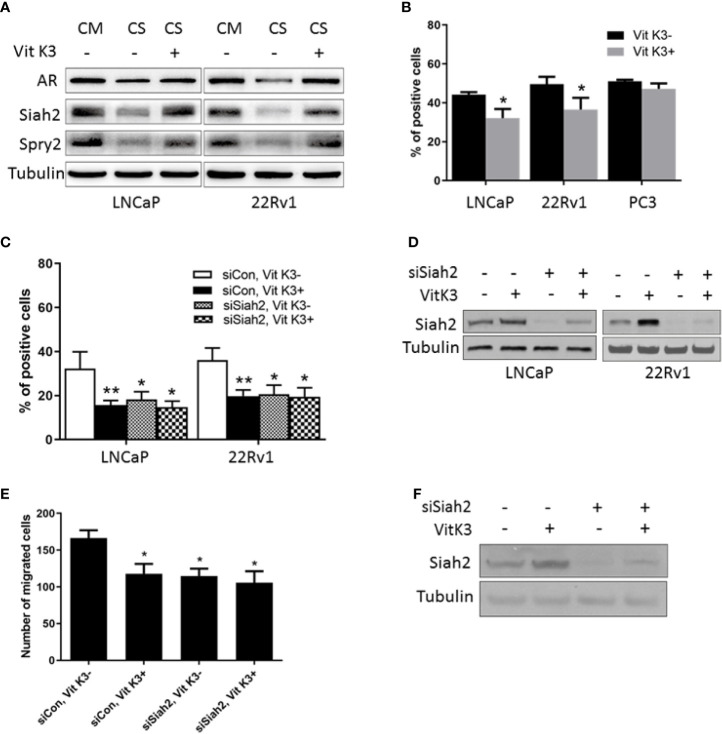
Vit K3 attenuated the effects of ADT on Siah2. **(A)** LNCaP and 22Rv1 cells were cultured in CS-FBS medium for 24 h, and then treated with Vit K3 (20 μM) for 24 h. Spry2, Siah2, and AR protein expression were detected by Western blotting. **(B)** LNCaP, 22Rv1, and PC3 cells were treated with Vit K3 (20 μM) for 24 h, and then treated with 10 μM BrdU for 5 h (LNCaP) or 2 h (22Rv1, PC3, and DU145). The BrdU assay was performed as described in the Materials and Methods. **(C)** LNCaP and 22Rv1 cells were transfected with siRNAs targeting Siah2 or control as indicated for 72 h, and then treated with Vit K3 (20 μM) or vehicle as indicated for 24 h. BrdU assays were performed as described in **(B)**. **(D)** The expression of Siah2 protein from cells treated as described for C was detected by Western blotting. **(E)** LNCaP cells were transfected with si-Siah2 or si-control as indicated, and then cultured in transwell chambers, as described in the Materials and Methods. Cells were then treated with Vit K3 (20 μM) or vehicle for 24 h, and subsequently were stained with crystal violet, after which the number of cells per field was quantified. **(F)** The expression of Siah2 protein from cells treated as described for E was detected by Western blotting. *P < 0.05; **P < 0.01.

Given that Siah2 plays a key role in the development of CRPC (12) and that ADT triggers its ligase activation, we next asked whether Siah2 could be blocked when PCa cells were treated by ADT. The only Siah2-targeting drug described so far is Vit K3 (also known as menadione), which was identified as a specific inhibitor of Siah2 ubiquitin ligase activity in a screen of U.S. Food and Drug Administration-approved therapeutic drugs (16). We next verified whether Vit K3 could attenuate the effect of ADT on Siah2. As shown in **Figure 4A**, Siah2 and Spry2 expression were decreased in LNCaP and 22Rv1 cells cultured in CS-FBS medium compared with those in complete medium, as described previously. Significantly, Vit K3 treatment increased Siah2 and Spry2 expression in CS-FBS medium (**Figure 4A**), which indicates that Vit K3 inhibited Siah2 activity.

We next evaluated the physiologic significance of Vit K3 on PCa cells. Treatment with 20 μM Vit K3 for 24 h significantly reduced proliferation of AR-positive LNCaP and 22Rv1 cells, but not of AR-negative PC3 cells (**Figure 4B**). Given that the effect of Siah2 on PCa cell proliferation was AR-dependent, we argue that the inhibitory effect of Vit K3 on PCa cell growth is Siah2- and AR-dependent. To further verify the role of Siah2, siRNAs targeting Siah2 were transfected into LNCaP and 22Rv1 cells. Silencing Siah2 and Vit K3 treatment independently resulted in reduced PCa cell proliferation, but Vit K3 treatment in Siah2-knockdown cells did not further reduce cell proliferation (**Figures 4C, D** **)**. Similarly, LNCaP cell motility was inhibited following Vit K3 treatment or Siah2 silencing, but no further changes were observed in Siah2-knockdown cells treated with Vit K3 (**Figures 4E, F** **)**. Collectively, these data support a model in which Vit K3 inhibits the growth and motility of PCa cells through a mechanism that involves inhibition of Siah2 activity.

### ADT Combined With Vit K3 Therapy Delayed the Formation of CRPC

**Figure 5:**
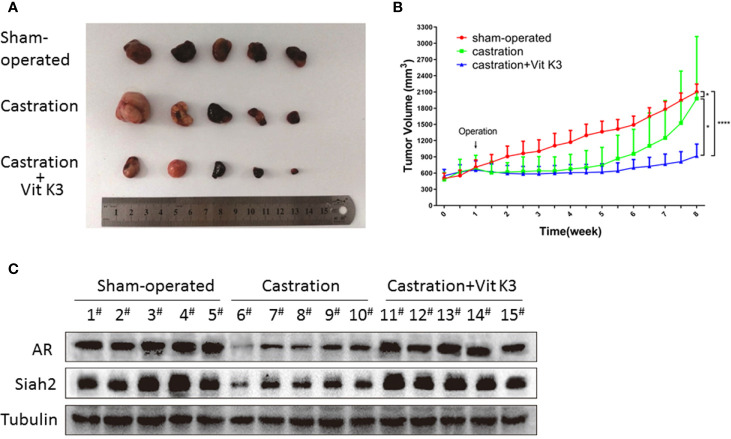
ADT combined with Vit K3 therapy delayed the formation of CRPC. **(A)** LNCaP cells were subcutaneously injected into BALB/c nude mice, and the animals were treated as described in the Materials and Methods. The tumor xenografts with different treatments are shown as indicated. **(B)** The growth curve of tumor xenografts. **(C)** Xenograft tumor tissue lysates were analyzed by Western blotting to detect AR and Siah2 expression in the sham-operated, castration, and castration+Vit K3 groups. *P < 0.05, ****P < 0.0001.

Having established that Vit K3 abolished the Siah2 activation triggered by ADT in PCa cells *in vitro*, we next sought to determine whether this mechanism was active *in vivo*. Therefore, we subcutaneously injected LNCaP cells into nude mice and monitored tumor development. Both castration and castration+Vit K3 treatment significantly reduced tumor volumes compared with the sham-treatment group. However, in the castration alone group, tumor volumes started to increase 3 weeks after castration and grew extremely fast 4 weeks later, which suggests the formation of CRPC. Strikingly, tumors treated with castration+Vit K3 only grew very slowly 4 weeks after treatment and still did not show fast growth after 8 weeks (**Figures 5A, B** **)**. Consistently, tumors derived from the Vit K3 treatment group were much smaller than those from the other two groups. Analysis of these tumors revealed significantly decreased Siah2 expression in response to castration, whereas Vit K3 treatment dramatically reversed expression Siah2, which was consistent with our *in vitro* findings (**Figure 5C**). These results strongly suggest that ADT+Vit K3 treatment may delay CRPC formation.

## Discussion

It is well established that the androgen-AR signaling axis plays a central role in CRPC. Most of the new drugs for CRPC approved by the FDA in recent years target AR signaling, including Abiraterone Acetate, an androgen synthesis inhibitor, and Enzalutamide and Apalutamide, second-generation AR antagonists. Although these second-generation antiandrogens show an overall survival benefit for advanced PCa, 20% to 40% of patients still do not respond to these therapies, and among the patients that do respond, resistance will eventually occur (17–19). Thus, the mechanisms of castration resistance need to be fully elucidated, as this will identify additional targets to treat and/or prevent CRPC.

Siah2 is an E3 ubiquitin ligase that targets AR and is thought to play an important role in the development of CRPC by targeting a select pool of chromatin-bound ARs that control the growth, survival, and tumorigenic capacity of PCa cells, especially under conditions of androgen deprivation (12). Interestingly, only small sets of metastatic PCa or CRPC showed a moderate 1.5- to 2-fold increase in Siah2 mRNA (12) These observations suggest that increased Siah2 transcription may not be the primary mechanism underlying the increased Siah2 activity observed in PCa tissues (15). Siah2 is very unstable because of self-ubiquitination and auto-degradation. Like other Ring finger E3 ubiquitin ligases, the ligase activity of Siah2 is reflected by its protein stability. Several factors, including the deubiquitinating enzymes USP13, AKR1C3, and DHX15 have been reported to stabilize Siah2 expression, by inhibiting its E3 ligase activity (14, 15, 20).

Here, we report that AR significantly stabilizes Siah2 protein expression and decreases its ligase activity in PCa cells. Notably, androgens extended the half-life of endogenous Siah2 to 5 h, which is much longer than the half-life in the presence of any of the other factors that have been reported to stabilize Siah2 (15, 20). These findings suggest that AR is a strong inhibitor of Siah2 ligase activity in PCa cells. As expected, ADT decreased Siah2 expression and enhanced its ligase activity. Importantly, we tested Siah2 expression in clinical PCa samples from the same patients before and after ADT, and found remarkably reduced Siah2 expression in response to ADT. These data indicate that during ADT, Siah2 is continuously activated in PCa. Based on these findings, we conclude that AR and Siah2 potentially form a positive regulatory loop in PCa, in which Siah2 mediates the ubiquitination-proteasomal degradation of a select pool of AR, whereas AR inhibits Siah2 ligase activity. ADT breaks the balance of these two proteins, which results in continuous Siah2 activation, which subsequently leads to CRPC.

Siah2 has three likely sites for intervention—interfering with its E3 ubiquitin ligase activity, the SBD domain, and the Siah–Siah dimerization domain (13). We demonstrated that AR reduced Siah2 auto-ubiquitination and increased Spry2 expression, a classic Siah2 substrate, which indicates that the mechanism through which AR stabilizes Siah2 is by blocking its E3 ligase activity. Co-immunoprecipitation studies revealed two domains of Siah2—the SBD and central RING domain/zinc finger domains—interacted with AR, consistent with the results of Qi et al. (12). We hypothesize that AR binding to the SBD is degraded as substrate, whereas AR binding to the central domain blocks the E3 ubiquitin ligase activity of Siah2. However, future structural studies will enable better assessment of the precise effects of AR on Siah2.

Vit K3 is a quinone used with cancer chemotherapeutics. Vit K3 and its analogs have been showed anticancer activities in several types of cancer including prostate cancer, breast cancer, melanoma and liver cancer *in vitro* and *in vivo* (16, 21, 22). Although the main biological effects on cancers are attributed to its role in the redox cycle and arylating nucleophilic substrates, Vit K3 has been identified as a specific inhibitor of Siah2 that inhibits both arms of the Siah2 downstream signaling network, the Ras/MAPK pathway and the hypoxic response pathway independent of reactive oxygen species. In this study, we showed that Vit K3 could abolish ADT-triggered Siah2 activation in PCa cells. Interestingly, we found that Vit K3 inhibited the growth of AR-positive LNCaP and 22Rv1 cells, but not AR-negative PC3 cells, which is consistent with its role of inhibiting Siah2 on PCa cells (12). Additionally, Vit K3 did not inhibit the growth or migration of Siah2-KD cells. Thus, we conclude that Vit K3 blocks PCa cell proliferation and motility at least partially by inhibiting Siah2.

Given that Siah2 plays a pivotal role in CRPC and that ADT triggers Siah2 activation in PCa cells, we hypothesized that ADT combined with a Siah2 inhibitor could block or delay the occurrence of CRPC. Strikingly, we demonstrated *in vivo* that ADT+Vit K3 treatment delayed the formation of CRPC and dramatically inhibited the growth of tumor xenografts compared with ADT alone. Importantly, analyses of these tumors suggested that Vit K3 delayed CRPC by inhibiting Siah2 activation.

In summary, this study provides new insights into the regulation of Siah2 in PCa. AR was identified as an inhibitor of Siah2. Because Siah2 is an E3 ubiquitin ligase for AR, we conclude that AR and Siah2 form a positive regulatory loop in PCa. ADT inhibits AR signaling, resulting in continuous Siah2 activation, which contributes to CRPC. Thus, ADT combined with Vit K3 may be a potential novel approach to delay the occurrence of CRPC.

## Data Availability Statement

The original contributions presented in the study are included in the article/**Supplementary Material**. Further inquiries can be directed to the corresponding authors.

## Author Contributions

YJ: conceptualization, investigation, supervision, writing the original draft, reviewing, and editing the manuscript. WG: methodology, investigation, reviewing, and editing the manuscript. TY: methodology, software, investigation, and writing the original draft. DZ: formal analysis, software, investigation, reviewing, and editing the manuscript. YS, DC, JJ, BH, HL, and SX: formal analysis, reviewing, and editing the manuscript. ZW: reviewing and editing the manuscript. All authors contributed to the article and approved the submitted version.

## Funding

This work was supported by the National Natural Science Foundation of China (81872098), the National Natural Science Foundation of China (81502212), and the Science and Technology Commission of Shanghai Municipality, China (18ZR1430600).

## Conflict of Interest

The authors declare that the research was conducted in the absence of any commercial or financial relationships that could be construed as a potential conflict of interest.

## Supplementary Material

Supplementary Figure 1 Androgens stabilize Siah2 protein. **(A)** LNCaP cells were cultured in CS for 24 h, subsequently treated with different dose of DHT or flutamide (5μM) as indicated for another 24 h, and AR and Siah2 protein expression were detected by Western blotting. **(B)** LNCaP and PC3 cells were cultured in CS for 24 h, subsequently treated with 10 nM DHT or vehicle for 24 h. AR and Siah2 protein expression were detected by Western blotting.

Supplementary Figure 2 AR inhibits Siah2 self-ubiquitination and decreases its E3 ligase activity. **(A)** 293 cells were transfected with Flag-Siah2, GFP-AR and Myc-Spry2 as indicated for 48 h and then treated with cycloheximide (CHX, 50 μg/ml) for 1, 3, 6 h and cell lysates were subjected to Western blotting.
