# Supplementary figures and images for: Targeting ADT-Induced Activation of the E3 Ubiquitin Ligase Siah2 to Delay the Occurrence of Castration-Resistant Prostate Cancer
Source: Front Oncol. 2021 Apr 16;11:637040. doi: 10.3389/fonc.2021.637040 (PMC8085430; doi:10.3389/fonc.2021.637040)

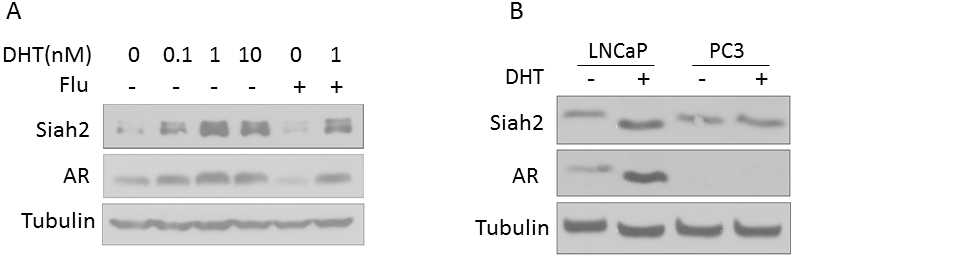

Supplement: Supplementary Figure 1 — Androgens stabilize Siah2 protein. (A) LNCaP cells were cultured in CS for 24 h, subsequently treated with different dose of DHT or flutamide (5μM) as indicated for another 24 h, and AR and Siah2 protein expression were detected by Western blotting. (B) LNCaP and PC3 cells were cultured in CS for 24 h, subsequently treated with 10 nM DHT or vehicle for 24 h. AR and Siah2 protein expression were detected by Western blotting. [file Image_1.tif]

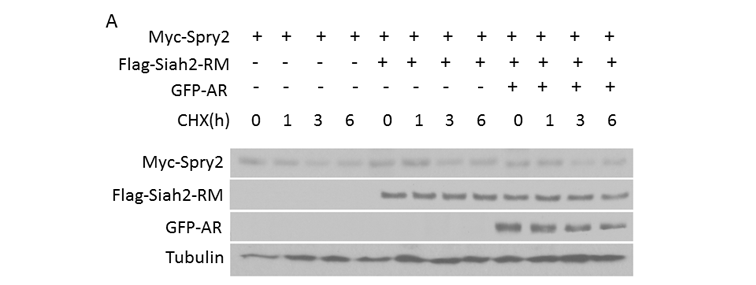

Supplement: Supplementary Figure 2 — AR inhibits Siah2 self-ubiquitination and decreases its E3 ligase activity. (A) 293 cells were transfected with Flag-Siah2, GFP-AR and Myc-Spry2 as indicated for 48 h and then treated with cycloheximide (CHX, 50 μg/ml) for 1, 3, 6 h and cell lysates were subjected to Western blotting. [file Image_2.tif]
